# Ventricular stabilization with a customized decellularized cardiac ECM-based scaffold after myocardial infarction alters gene expression in a rodent LAD-ligation model

**DOI:** 10.3389/fbioe.2022.896269

**Published:** 2022-09-23

**Authors:** Hug Aubin, Lenard Rath, Alexandra Vey, Vera Schmidt, Mareike Barth, Elvira Weber, Artur Lichtenberg, Payam Akhyari

**Affiliations:** ^1^ Department of Cardiac Surgery, Medical Faculty and University Hospital Düsseldorf, Heinrich-Heine-University Düsseldorf, Duesseldorf, Germany; ^2^ Research Group 3D Cardiovascular Regenerative Medicine and Tissue Engineering (CURE 3D), Department of Cardiac Surgery, Medical Faculty and University Hospital Düsseldorf, Heinrich-Heine-University Düsseldorf, Düsseldorf, Germany

**Keywords:** myocardial infarction, decellularized extracellular matrix, gene expression, tissue engineering, cardiac regeneration

## Abstract

**Objectives:** Decellularized extracellular matrix (dECM) is increasingly used in a wide range of regenerative medicine applications and may also offer the potential to support injured myocardium. Here, we evaluated the myocardial gene expression pattern after myocardial infarction (MI) in a standardized rodent LAD-ligation model with and without ventricular stabilization with a customized, cardiac dECM-based scaffold (cdECM).

**Methods:** MI was induced in male Wistar rats by standard LAD-ligation and confirmed 14 days post-intervention by echocardiographic parameters (FAS<40%). Cardiac ECM from donor rats was used to generate individual cdECM-scaffolds (tissue engineered myocardial sleeve, TEMS), which were epicardially implanted after confirmed MI for ventricular stabilization. After 4 and 8 weeks heart function was assessed by echocardiography, rats were sacrificed and explanted hearts were analyzed. In addition to histological analysis, standardized anterior left ventricular wall myocardial tissue samples were assessed by quantitative real-time PCR evaluating the specific gene expression pattern for immunomodulatory (IL-10, TGFBR2, TNFα), pro-angiogenic (VEGFA, FGF2, PGF, PDGFB), pro-survival (HGF, SDF1, IGF1, AKT1), remodeling-associated (TIMP1, MMP2, MMP9) and infarction-specific (NPPA, NPPB) markers.

**Results:** Ventricular stabilization led to integration of the TEMS-scaffold into the myocardial scar with varying degrees of cellular infiltration, as well as significantly improved echocardiographic parameters demonstrating attenuation of maladaptive cardiac remodeling. Further, TEMS implantation after MI altered the myocardial gene expression pattern. Differences in gene expression were most striking after 4 weeks with significantly reduced expression of NPPA (0.36 ± 0.26 vs 0.75 ± 0.40; *p* < 0.05), NPPB (0.47 ± 0.25 vs 0.91 ± 0.429; *p* < 0.01), TGFBR2 (0.68 ± 0.16 vs 0.90 ± 0.14; *p* < 0.01) and PDGFB (0.81 ± 0.13 vs 1.06 ± 0.14; *p* < 0.01) as well as increased expression of IL-10 (5.93 ± 5.67 vs 1.38 ± 0.60; *p* < 0.05), PGF (1.48 ± 0.38 vs 1.09 ± 0.25; *p* < 0.05) and IGF1 (1.67 ± 0.70 vs 1.03 ± 0.42; *p* < 0.05). However, after 8 weeks differences in the gene expression patterns of remodeling-associated, and pro-angiogenic markers could still be observed between groups.

**Conclusion:** Ventricular stabilization via TEMS implantation after MI did not only led to biological integration of the cdECM-scaffolds into the host tissue and improved functional cardiac parameters, but also altered 4 and 8 week gene expression of infarcted myocardium, possibly contributing to reducing chronic deteriorating effects while increasing the potential for myocardial regeneration.

## Introduction

Decellularized extracellular matrix (dECM) based scaffolds are increasingly used in a wide range of regenerative medicine applications, as they maintain the complex microarchitecture and ultracomposition of native tissues and hence provide ideal cues for tissue regeneration (Taylor et al., 2018). In the cardiovascular field, dECM based scaffolds have already been successfully employed in many pre-clinical and clinical models, with decellularized heart valves being at the verge of changing paradigms of heart valve therapy (Horke et al., 2020). Decellularized ECM scaffolds may also offer great potential for regeneration and repair of damaged myocardium, as composition and organization of cardiac ECM - critical for cell support and tissue function–can be reproduced by decellularization of cardiac ECM (Bejleri and Davis, 2019). Although whole-heart tissue engineering approaches have impressively shown the prospects of cardiac-based ECM scaffolds, the generation of a bioartificial heart replacement is still very far afield due to biological and technical complexity (Tang-Quan et al., 2018). Hence, efforts in the field should concentrate on exploring strategies to support, repair and/or regenerate the damaged myocardium instead of replacing it. Such therapies are strongly warranted, as for today, the only viable options for the treatment of end-stage heart failure (HF) consist in the implantation of a permanent mechanical assist device or heart transplantation (McDonagh et al., 2021). Other surgical and experimental approaches that have been explored in the past, such as surgical cardiomyoplasty or stem cell therapy, have been mostly abandoned due to disappointing outcomes (Hetzer et al., 2021).

Here, we introduce a new therapy concept using a customized, decellularized cardiac ECM-based scaffold (cdECM-scaffold) in form of a tissue engineered myocardial sleeve (TEMS) for ventricular stabilization after myocardial infarction, hypothesizing that this might stimulate myocardial regeneration, hinder adverse cardiac remodeling and improve cardiac hemodynamics. This proof-of-concept study focuses on the effect on myocardial gene-expression after ventricular stabilization via TEMS in a standardized rodent LAD-ligation *in vivo* model.

## Materials and methods

All animal experiments and surgical procedures were performed in compliance with the Guide for the Care and Use of Laboratory Animals as published by the US National Institutes of Health (NIH Publication 85-23, revised 1996) and approved by the local animal care committees (Registration No. 84-01.04.2012. A335).

### Whole-heart decellularization

Donor hearts of Wistar rats (male, 350–450 g) were explanted and perfusion based *in toto* heart decellularization was performed via standardized automated software-controlled coronary perfusion with decellularization agents as previously described (Akhyari et al., 2011). Briefly, decellularization protocol consisted of perfusion with 0.5% sodium dodecyl sulfate (SDS; CarlRoth) and 0.5% desoxycholic acid (DCA; Amresco) for 48 h with subsequent perfusion with deionized water and phosphate-buffered saline (PBS; Gibco) for 24 and 72 h, respectively. Decellularized whole-hearts were stored in PBS supplemented with penicillin/streptomycin at 4°C before further processing.

### Tissue engineered myocardial sleeve

**FIGURE 1:**
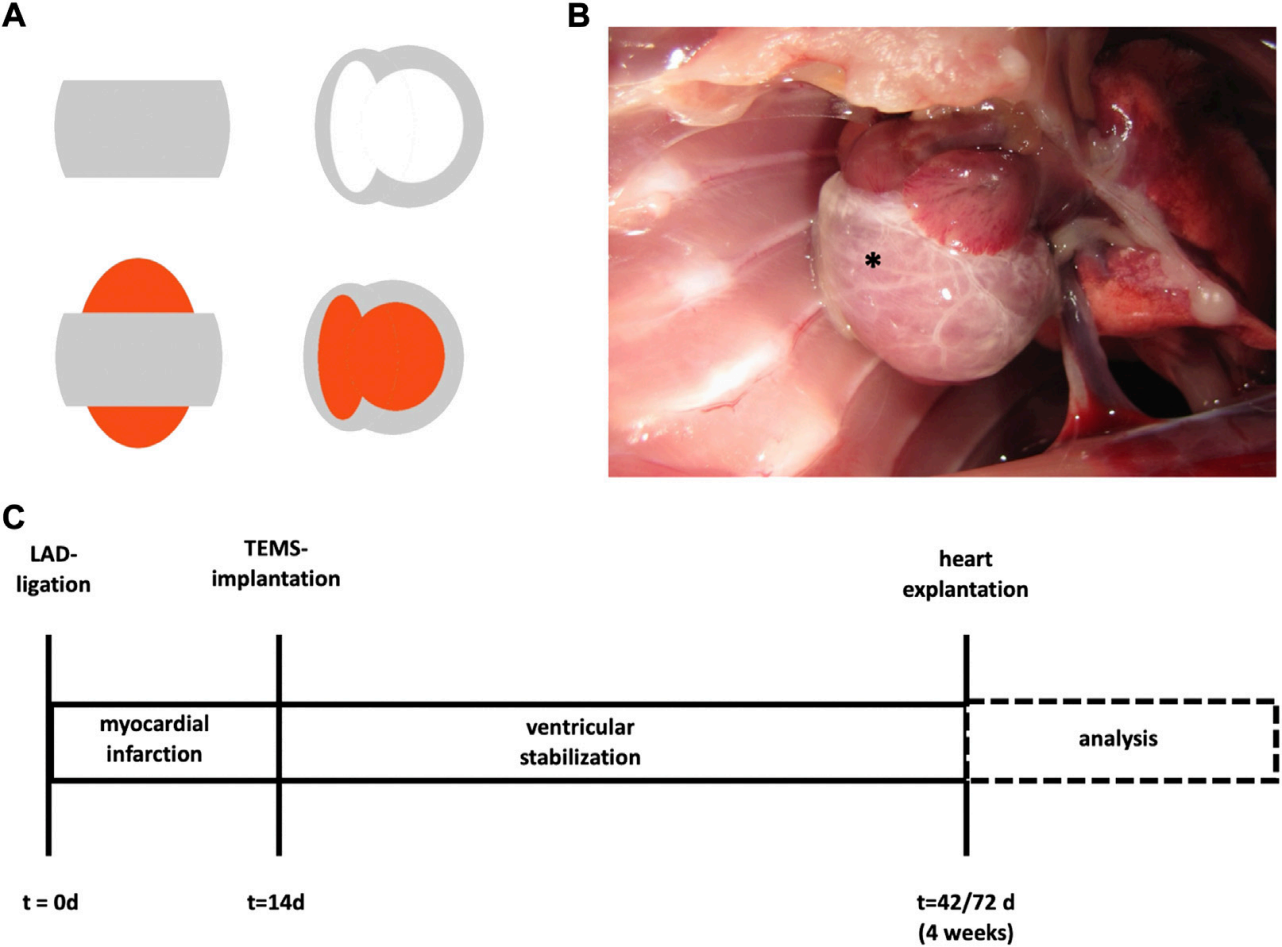
TEMS schematics and timeline surgical protocol. **(A)** Schematics of ventricular stabilization via TEMS. Grey, cdECM scaffold; red, native heart. **(B)** Macroscopic image of ventricular stabilization via TEMS in rodent model. *, TEMS. **(C)** Myocardial infarction was induced by LAD-ligation in a standard rodent model and after 14 days ventricular stabilization via TEMS implantation was performed. Hearts were explanted and analyzed after 4 or 8 weeks. LAD, left anterior descending; TEMS, tissue engineered myocardial sleeve.

Customized, decellularized cardiac ECM-based scaffolds (cdECM-scaffolds) where obtained through standardized microsurgical dissection of the decellularized whole-hearts. Therefore, the atria including the atrioventricular heart valves, the septum and the apex of the decellularized whole-heart were removed, leaving a myocardial sleeve consisting of the free walls of the right and left ventricle (TEMS; tissue engineered myocardial sleeve) (Figure 1A). Dissected tissue was analyzed for removal of cellular components and degree of ECM conservation, as already previously published (Aubin et al., 2013a). TEMS were stored in PBS supplemented with penicillin/streptomycin at 4°C before further processing.

### Surgical protocol

In order to evaluate the biological effects of ventricular stabilization with a cdECM-scaffold after myocardial infarction we used a standard rodent LAD (left anterior descending coronary artery)-ligation model with Wistar rats (from an in-house breed of the local animal care facility) as animal model. Fourteen days after LAD-ligation the cdECM-scaffold in form of a TEMS was then epicardially implanted and left *in situ* for either 4 or 8°weeks, after which the experimental animal was euthanized, the heart explanted and analyzed **(** Figure 1C **).**

LAD-ligation induced myocardial infarction was confirmed by standard echocardiographic parameters (Galrinho et al., 2015). Only animals which presented with a FAS (fractional area shortening) of the left ventricle <40% 14 days after LAD-ligation entered the LAD-ligation group. In cases where FAS remained >40% after LAD-ligation or the explantation timepoint was not reached due to mortality or morbidity experimental animals dropped out of further analysis. As control groups respective sham operations for the LAD-ligation and the TEMS implantation were performed, hence, experimental groups as shown in Table 1 were analyzed.

**TABLE 1:** Experimental groups, timepoints and rationale.

Experimental group^a^	timepoints	rationale
-LAD/-TEMS	4/8 weeks	control (native heart)
-LAD/+TEMS	4/8 weeks	effect TEMS on healthy heart
+LAD/-TEMS	4/8 weeks	control (infarcted heart)
+LAD/+TEMS	4/8 weeks	effect TEMS on infarcted heart

a -LAD, respective sham operation; +LAD, myocardial infarction via LAD-ligation; -TEMS, respective sham operation; +TEMS, ventricular stabilization via TEMS, implantation.

#### Rodent LAD-ligation model

For induction of myocardial infarction, a standard LAD-ligation model was used (Gao et al., 2010). Wistar rats (male, 200–250 g), fed *ad libitum* with standard rat chow, were used as a rodent animal model. For LAD-ligation, rats were anesthetized with 2.0–2.5% isoflurane, orotracheally intubated and machine ventilated with room air supplemented with oxygen using a small animal ventilator (tidal volume, 10 ml/kg). The respiratory rate was adjusted to maintain the partial pressure of CO_2_ within physiological limits. Body temperature was maintained at 37°C–37.5°C with a heating pad. Baseline echocardiographic parameters were recorded, and afterwards a lateral left-sided thoracotomy was performed. After pericardiotomy, the heart was exposed, the left anterior descending coronary artery (LAD) was identified and a 10–0 monofilament non-absorbable polypropylene suture (Prolene, Ethicon, Norderstedt, Germany) was looped around the LAD and ligated. Coronary artery occlusion was immediately verified by blanching and cyanosis of the myocardial area at risk (experimental group: +LAD). After LAD-ligation the thoracotomy was closed in layers. For LAD-ligation sham operation (experimental group: LAD) the suture was only looped around a major branch of the LAD and removed, without ligation.

#### TEMS implantation

TEMS were implanted 14 days after LAD-ligation (or sham operation). Therefore, rats were anesthetized, orotracheally intubated and machine ventilated as described above. Echocardiographic parameters were recorded in order to evaluate efficacy of prior LAD-ligation. Afterwards thoracotomy was performed using the surgical access site from the prior operation in order to expose the heart. The TEMS was slipped over a copped 2 ml syringe (B. Braun, Melsungen, Germany), whose opening was used to apply gentle suction force on the apex, luxate the heart ventrally and pull the TEMS over the native heart. TEMS was aligned anatomically correct (left and right ventricle aligned accordingly) and fixated with four stitches using 10-0 Prolene suture (Ethicon, Raritan, NJ, US) so that it covered most of the surface of both ventricles (experimental group: +TEMS) (Figure 1B). After implantation, the thoracotomy was closed again in layers. For TEMS implantation sham operation (experimental group: TEMS) the native heart was luxated and four holding sutures were placed accordingly.

#### Heart explantation

Four and 8 weeks after TEMS implantation or sham operation experimental animals were narcotized and echocardiographic parameters were recorded. After euthanasia the heart was explanted via median sternotomy and processed depending on subsequent tissue analysis.

#### Echocardiographic assessment

Echocardiographic assessment was performed on anesthetized animals at baseline, 14, 42 and 70 days, according to the experimental protocol, using a Philips HDX11 ultrasonography system equipped with a 15- MHz probe (Philips, Hamburg, Germany). Following parameters were evaluated in a standardized analysis: left ventricular end-diastolic diameter (LVEDD), ejection fraction (EF) and fractional area shortening (FAS) of the left ventricle (Galrinho et al., 2015)**.**

### Tissue analysis

#### Histology

For orientating histological analysis, explanted hearts were dissected into standardized slices, fixed in a 4% buffered formaldehyde solution (Roth, Karlsruhe, Germany) and processed via cryo-sectioning (CM 1950; Leica, Wetzlar, Gemany) using standard protocols. Frozen sections of 6 mm were then stained with hematoxylin and eosin (H&E) and Movat’s pentachrom staining according to standard protocols, and then visualized using a transmission light microscope (DM 2000; Leica, Wetzlar, Gemany).

#### Gene expression

For evaluation of gene expression, freshly explanted hearts were dissected into standardized probes separating the TEMS from the native heart (heart: anterior and posterior wall of the left ventricle; TEMS in projection to the anterior wall of the native heart). Probes were then snap-frozen in liquid nitrogen and pulverized. RNA was isolated of processed tissue samples using the RNeasy Mini Kit (Quiagen, Hilden, Germany) according to the manufacturer’s protocol. RNA quality was analyzed by automated electrophoresis (Agilent 2100 Bioanalyzer (Agilent, Santa Clara, California, United States). Isolated RNA was subjected to cDNA synthesis using the QuantiTect Reverse Transcription Kit (Quiagen, Hilden, Germany) according to the manufacturer’s protocol using a real time cycler (StepOne Plus, Applied Biosystems, Foster City, United States). Synthesized cDNA samples then underwent real-time-polymerase chain reaction (qRT-PCR) to assess the expression levels of specific immunomodulatory (IL 10, TGFBR2, TNFα), pro-angiogenic (VEGFA, FGF2, PGF, PDGFB), pro-survival (HGF, SDF1, IGF1, AKT1), remodeling-associated (TIMP1, MMP2, MMP9) and infarction-specific (NPPA, NPPB) genes using top2b as well as beta2‐microglobulin as respective house-keeping genes. Primers were selected from the National Center for Biotechnology Information (NCBI) database (Supplementary Table S1). Recorded values were processed and analyzed using the ∆∆CT method, representing relative increase or decrease of expression levels directly by evaluating the ratios between experimental samples and their corresponding controls. For the 4 weeks experimental groups at least eight biological replicates and for the 8 week experimental group at least six biological replicates were used. Data are presented for both housekeeping genes.

#### Statistics

All values are presented as mean −/+ standard deviation of the mean for all continuous variables. For direct group comparisons at one single time point, Student’s t-tests with or without Welch’s correction or Mann–Whitney U tests were performed. Statistical significance was assumed if *p*-values were lower than 0.05. Data analysis was conducted with GraphPad Prism v5.04 (GraphPad Software, San Diego, CA).

## Results

### Surgical outcome

Ventricular stabilization with a cdECM-based scaffold via TEMS-implantation either with or without LAD-ligature was performed in 56 animals. Peri- and postoperative mortality was 35.7% and mostly due to perioperative complications either related to the anaesthesia or the surgical procedure. Mortality after successful TEMS implantation was neglectable, with 36 animals (64.3%) reaching the end of the experiment after 4 or 8 weeks. Together with the respective sham control groups 86 animals reached the endpoint and were analysed.

### Echocardiographic parameters

**FIGURE 2:**
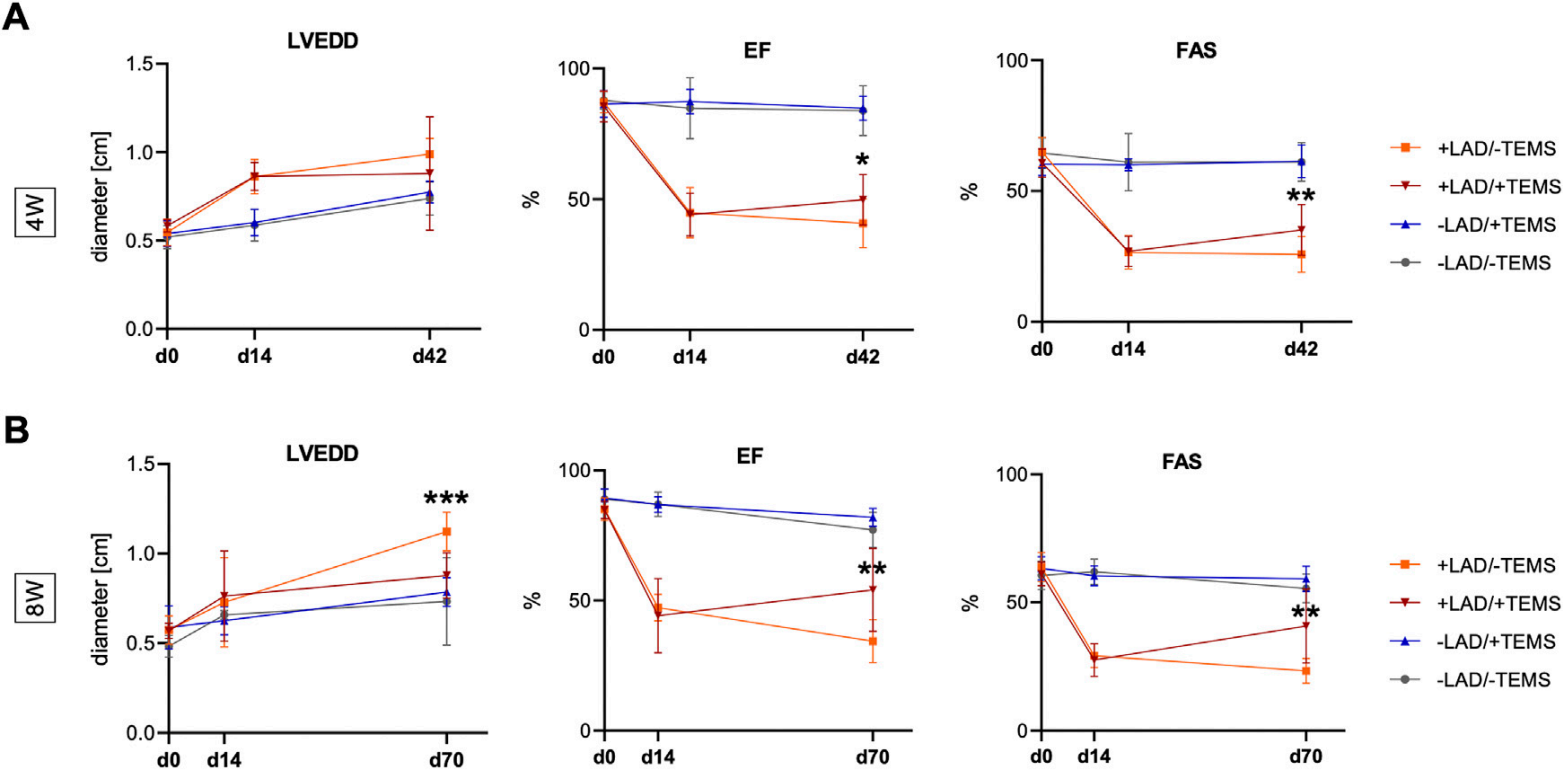
Echocardiographic parameters. Additional TEMS-implantation 14 days after LAD-ligature decreased LVEDD (left ventricular end-diastolic diameter) and improved left ventricular EF (ejection fraction) and FAS (fractional area shortening) after 4 and 8 weeks. **(A)** LVEDD, EF and FAS 14 days after LAD-ligature (+LAD) and 4 weeks after TEMS-implantation (+TEMS) or respective shame operations (-LAD; -TEMS), showing significantly improved EF and FAS in the +LAD/+TEMS as compared to the +LAD/-TEMS-group, and no significant differences between the -LAD/-TEMS and -LAD/+TEMS group. **(B)** LVEDD, EF and FAS 14 days after LAD-ligature and 8 weeks after TEMS-implantation or respective shame operations, showing significantly reduced LVEDD as well as improved EF and FAS in the +LAD/+TEMS-group as compared to the +LAD/-TEMS group, and no significant differences between the–LAD/-TEMS and–LAD/+TEMS group. Experimental groups: LAD/-TEMS, grey; -LAD/+TEMS, blue; +LAD/-TEMS, orange; +LAD/+TEMS, red. Data presented as mean ± SD; *, *p* < 0.05; **, *p* < 0.01; ***, *p* < 0.001).

As expected LAD-ligature led to a significant decrease in EF (84.8 ± 3.3% vs. 44.9 ± 9.2%, *p* < 0.0001) and a significant increase in LVEDD (0.59 ± 0.09 cm vs 0.86 ± 0.09 cm, *p* < 0.0001) after 14 days, as compared to the sham control group (Figure 2). Mean FAS in LAD-ligature animals entering the experimental groups was 26.5 ± 6.1%. Animals subsequently receiving an additional TEMS-implantation 14 days after LAD-ligature had by trend smaller LV diameters (0.88 ± 0.04 cm vs 0.99 ± 0.08 mm, *p* = 0.13) with however significant improved EF (49.8 ± 9.2% vs. 40.8 ± 9.0%, *p* < 0.05) as well as FAS (35.1 ± 9.1% vs. 25.8 ± 6.5%, *p* < 0.01), as compared to the respective shame control group (+LAD/-TEMS) after 4 weeks (Figure 2A). Eight weeks after TEMS-implantation LV diameters were significantly smaller (0.88 ± 0.12 cm vs 1.12 ± 0.10 mm, *p* < 0.0001) with further increase in EF (54.1 ± 14.7% vs. 34.4 ± 7.8%, *p* < 0.01) as well as LV-FAS (40.8 ± 13.3% vs. 23.3 ± 4.5%, p < 0.01), as compared to the respective shame control group (+LAD/-TEMS) (Figure 2B). TEMS-implantation in animals without myocardial infarction did not have any significant impact on LVEDD, EF nor FAS after 4 or 8 weeks, as compared to their respective sham control group (-LAD/-TEMS) (Figures 2A,B).

### Macroscopic appearance

**FIGURE 3:**
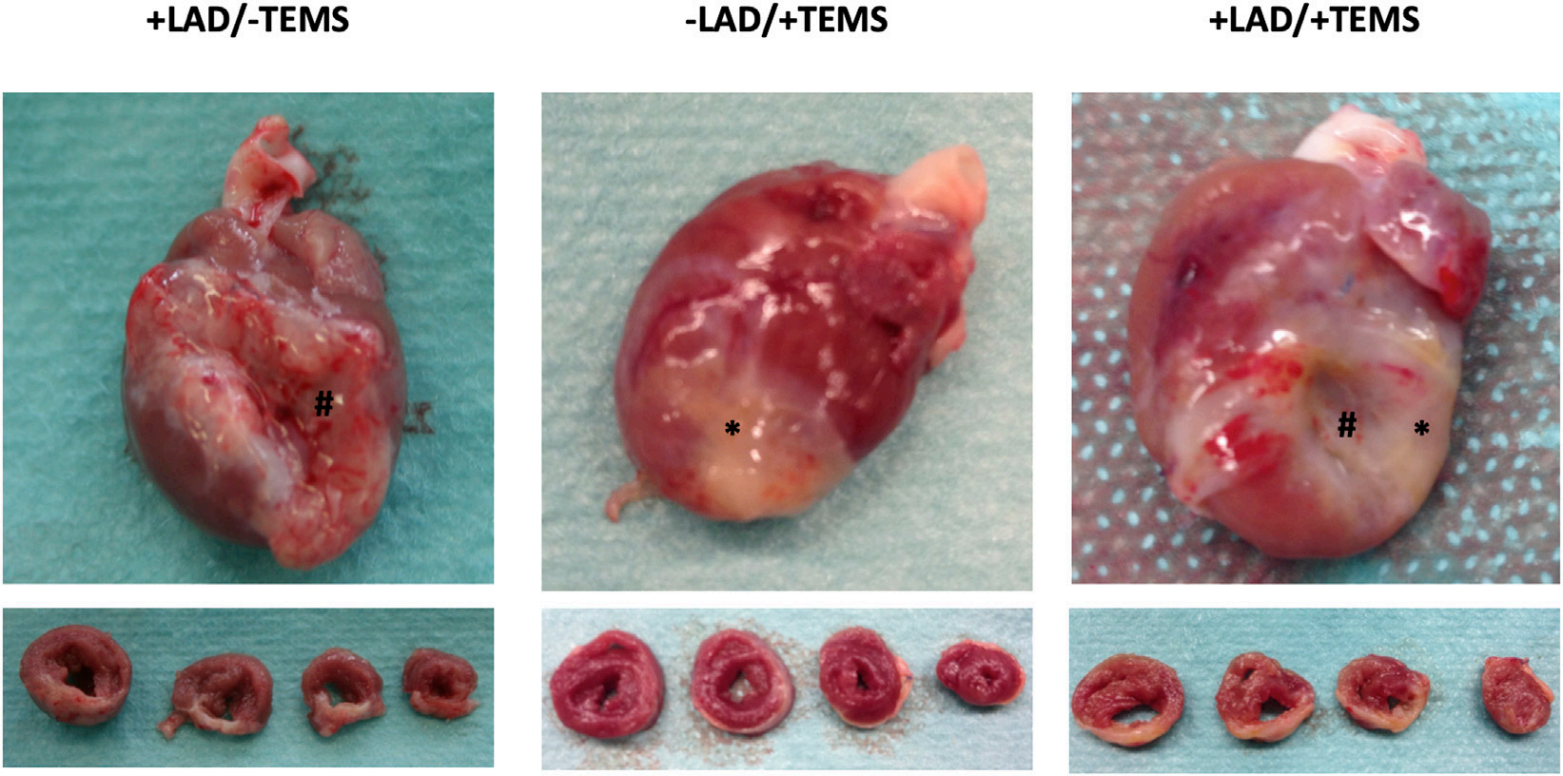
Macroscopic appearance of explanted heart specimens after LAD-ligation and TEMS implantation. Representative images of explanted whole heart and sliced specimens 4 weeks after either LAD-ligation, TEMS implantation and LAD-ligation plus TEMS-implantation. Region of myocardial infarction as wells TEMS scaffold was clearly distinguishable in all specimens. #, region of myocardial infarction; *, TEMS scaffold.

Representative images of explanted heart specimens 4 weeks after TEMS-implantation are shown in Figure 3. The area of myocardial infarction was clearly distinguishable in animals with LAD-ligature with aneurysm and scar tissue formation. In the explanted hearts the cdECM-based scaffold of the TEMS was still largely intact and could be identified around the whole circumference of the heart. After 4 and 8 weeks *in vivo* the TEMS scaffold had adhered to the epicardium and seemed to be especially integrated to the native tissue in the infarct area. TEMS scaffolds could however be mechanically removed from the heart for further analysis.

### Orientating histological analysis

**FIGURE 4:**
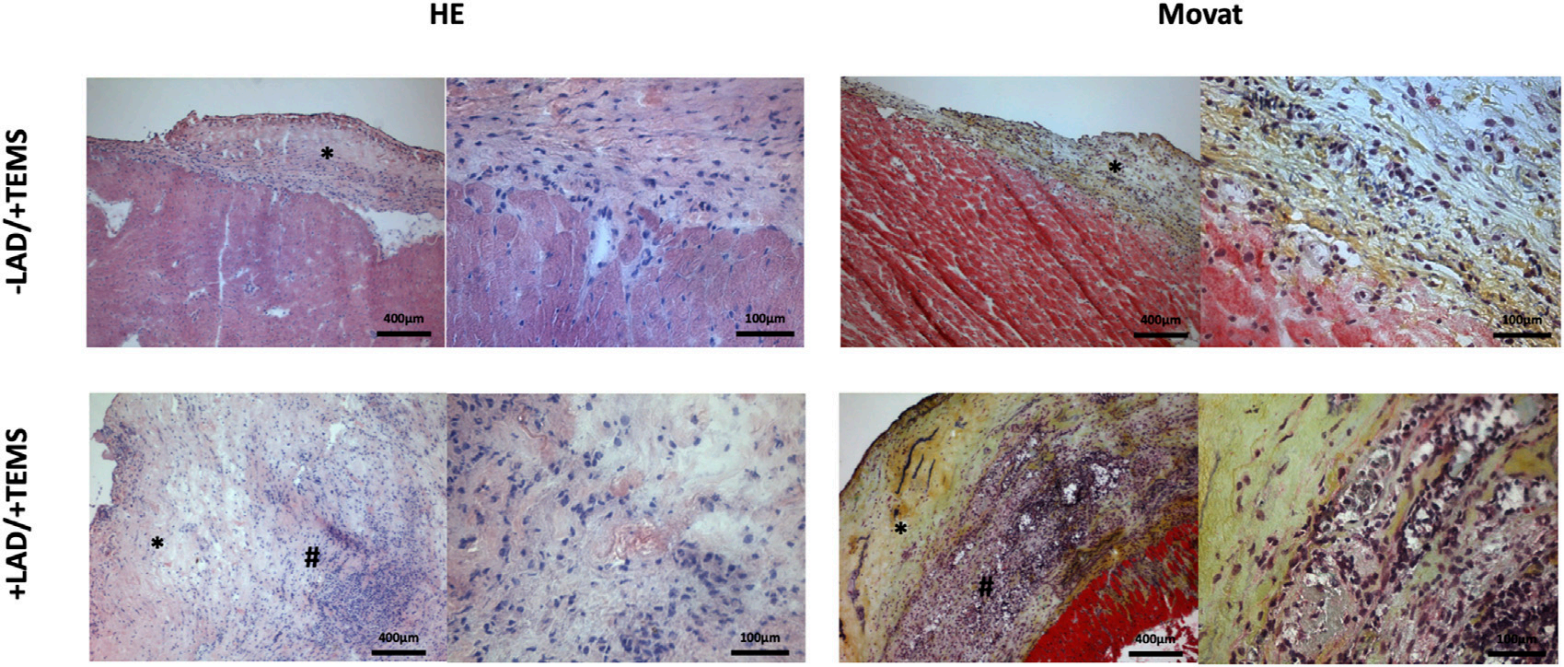
Orientating histological analysis. Representative images of HE and MOVAT staining of explanted hearts 4 weeks after ventricular stabilization via TEMS implantation with and without prior myocardial infarction via LAD-ligature, showing high degree of cellular infiltration of the TEMS scaffold (*) as well as integration of the TEMS scaffold into the infarcted zone (#) of the heart.

LAD-ligature led to classical histological findings after myocardial infarction with formation of granulation tissue, neovascularization and decreased cellularity (data not show). Ventricular stabilization via TEMS implantation led to biological integration of the TEMS scaffold to the native myocardium with native cells infiltrating the TEMS scaffold (Figure 4). As opposed to the non-infarcted hearts where the TEMS scaffold could still be clearly differentiated from the native myocardium on the left anterior wall, in hearts with prior LAD-ligature TEMS scaffolds highly integrated into the infarcted region without distinguishable boundaries between native myocardium and implanted tissue. Cellular infiltration into the TEMS scaffold was highly increased at the interface between native tissue and the cdECM, with areas of very high degree of cellular infiltration especially within the infarcted myocardial area. There were no gross differences in the histological analysis of hearts explanted either 4 or 8 weeks after TEMS implantation (data not shown).

### RNA concentration

Evaluation of TEMS tissue prior to implantation revealed no detectable RNA in the cdECM scaffolds. However, due to the *in vivo* cellular infiltration, the explanted TEMS tissue contained high degrees of RNA, ranging between 40% and 70% of the RNA concentration in the adjacent native myocardial tissue. In the TEMS tissue adjacent to the infarcted region of the heart, RNA concentration was 238.5 ± 102.8 ng/μl after 4 weeks and 161.04 ± 92.65 ng/μl after 8 weeks *in vivo*, as compared to 354.2 ± 92.0 ng/μl and 331.8 ± 128.5 ng/μl in the respective adjacent anterior wall of the LV.

### Gene expression

#### Myocardial tissue

As expected, myocardial infarction via LAD-ligation did significantly changed the expression pattern of the analyzed genes in the infarcted myocardial area of the left anterior ventricular wall after 4 as well as 8 weeks, as compared to the respective sham control group (-LAD) (Supplementary Table S2). In the adjacent, non-infarcted myocardial regions, LAD-ligation had no influence in the gene expression patterns (data not shown).

**FIGURE 5:**
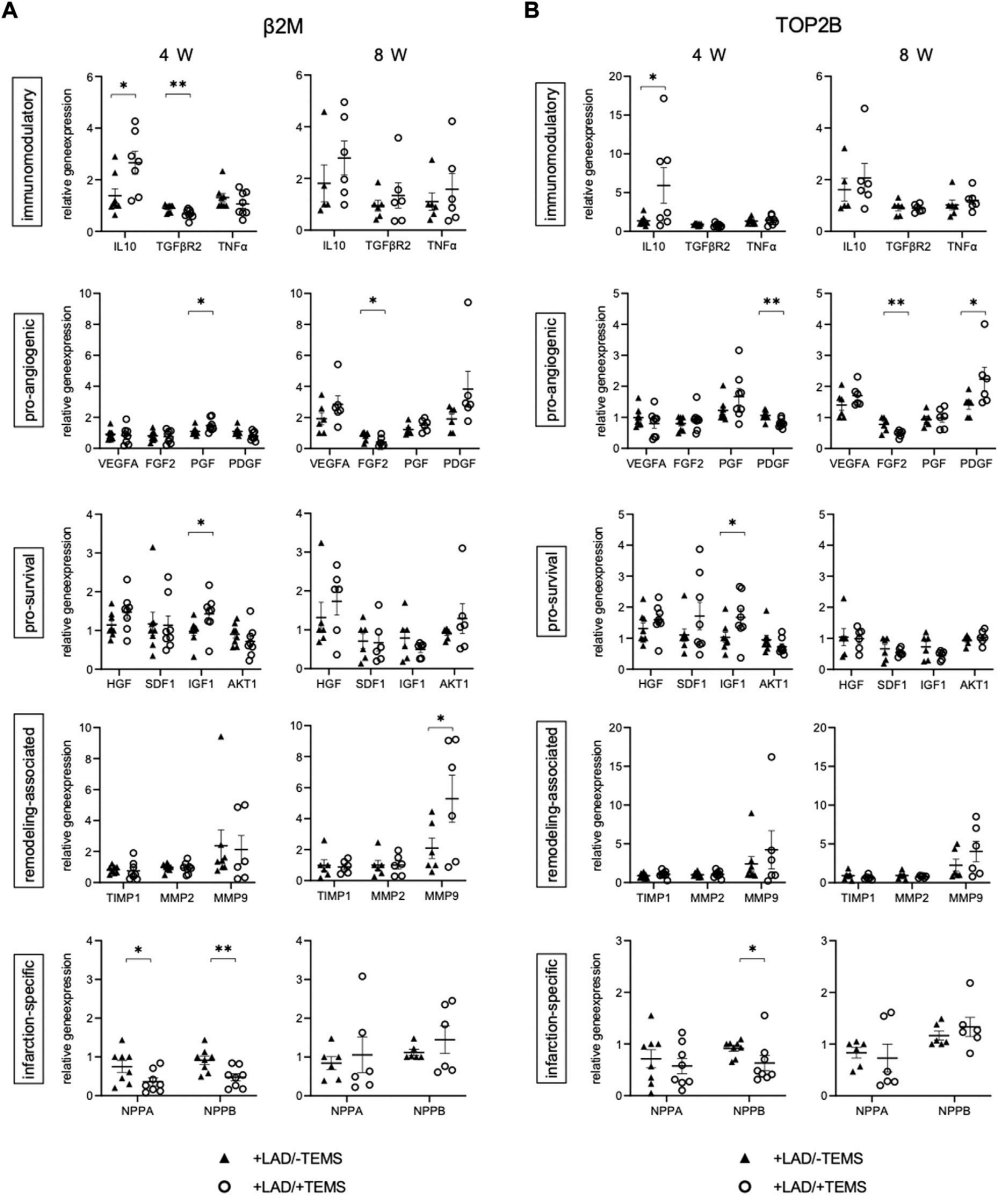
Gene expression after myocardial infarction with and without ventricular stabilization via TEMS implantation. Relative gene expression patterns of left anterior wall myocardial tissue samples after myocardial infarction via LAD-ligation (+LAD) and either 4 or 8 weeks of ventricular stabilization via TEMS implantation (+TEMS) or respective sham control (-TEMS). **(A)** Gene expression relative to β2M housekeeping gene. **(B)** Gene expression relative to TOP2B housekeeping gene. Data presented as mean ± SD; *, *p* < 0.05; **, *p* < 0.01; ***, *p* < 0.001.

Gene expression of the myocardial tissue within the infarcted region was further altered after ventricular stabilization via TEMS-implantation, as compared to the respective sham control group (+LAD/-TEMS) (Table 2, Figure 5). Differences in gene expression were most striking after 4 weeks with significantly reduced infarction-related expression of NPPA (0.36 ± 0.26 vs 0.75 ± 0.40; β2m; p < 0.05) and NPPB (0.47 ± 0.25 vs 0.91 ± 0.429; β2m; p < 0.01), increased expression of immunomodulatory IL-10 (5.93 ± 5.67 vs 1.38 ± 0.60; top2b; p < 0.05) and reduced TGFBR2 (0.68 ± 0.16 vs 0.90 ± 0.14; β2m; p < 0.01), increased expression of pro-angiogenic PGF (1.48 ± 0.38 vs 1.09 ± 0.25; β2m; p < 0.05) and reduced PDGFB (0.81 ± 0.13 vs 1.06 ± 0.14; top2b; p < 0.01) as well as increased expression of pro-survival IGF1 (1.67 ± 0.70 vs 1.03 ± 0.42; top2b; p < 0.05) after LAD-ligation and TEMS implantation as compared to LAD-ligation alone (Figures 5A,B). However, significant changes in the gene expression pattern could still be seen 8 weeks after TEMS implantation following LAD-ligature with reduced expression of pro-angiogenic FGF2 (0.48 ± 0.10 vs. 0.78 ± 0.21; top2b; p < 0.01) and increased PDGFB (2.24 ± 0.86 vs 1.41 ± 0.32; top2b; p < 0.05) as well as increased expression of remodeling-associated MMP9 (5.29 ± 3.41 vs 2.10 ± 1.47; β2m; p < 0.05) as compared to the respective control (Figures 5A,B).

**TABLE 2:** Relative changes in gene expression of the anterior left ventricular myocardial wall after ventricular stabilization via TEMS-implantation following myocardial infarction.

Gene group		relative changes in gene expression*			
		4 weeks		8 weeks	
housekeeping		TOP2B	β2M	TOP2B	β2M
**immunomodulatory**	IL 10	↑*	↑*	↑	↑
	TNF α	↑	↓	↑	↑
	TGFBR2	↓	↓**	↓	↑
**pro-angiogenic**	VEGFA	↓	↓	↑	↑
	FGF2	↑	↓	↓**	↓*
	PGF	↑	↑*	↑	↑
	PDGFB	↓**	↓	↑*	↑
**pro-survival**	HGF	↑	↑	↓	↑
	SDF1	↑	↓	↓	↓
	IGF1	↑*	↑*	↓	↓
	AKT1	↓	↓	↑	↑
**remodeling-associated**	MMP2	↑	↓	↓	↑
	MMP9	↑	↓	↑	↑*
	TIMP1	↑	↓	↓	↓
**infarction-specific**	NPPA	↓	↓*	↓	↑
	NPPB	↓*	↓*	↑	↑

Relative changes to anterior left ventricular myocardial wall of respective sham control group. *, *p* < 0.05; **, *p* < 0.01; ***, *p* < 0.001.

#### TEMS scaffold

**FIGURE 6:**
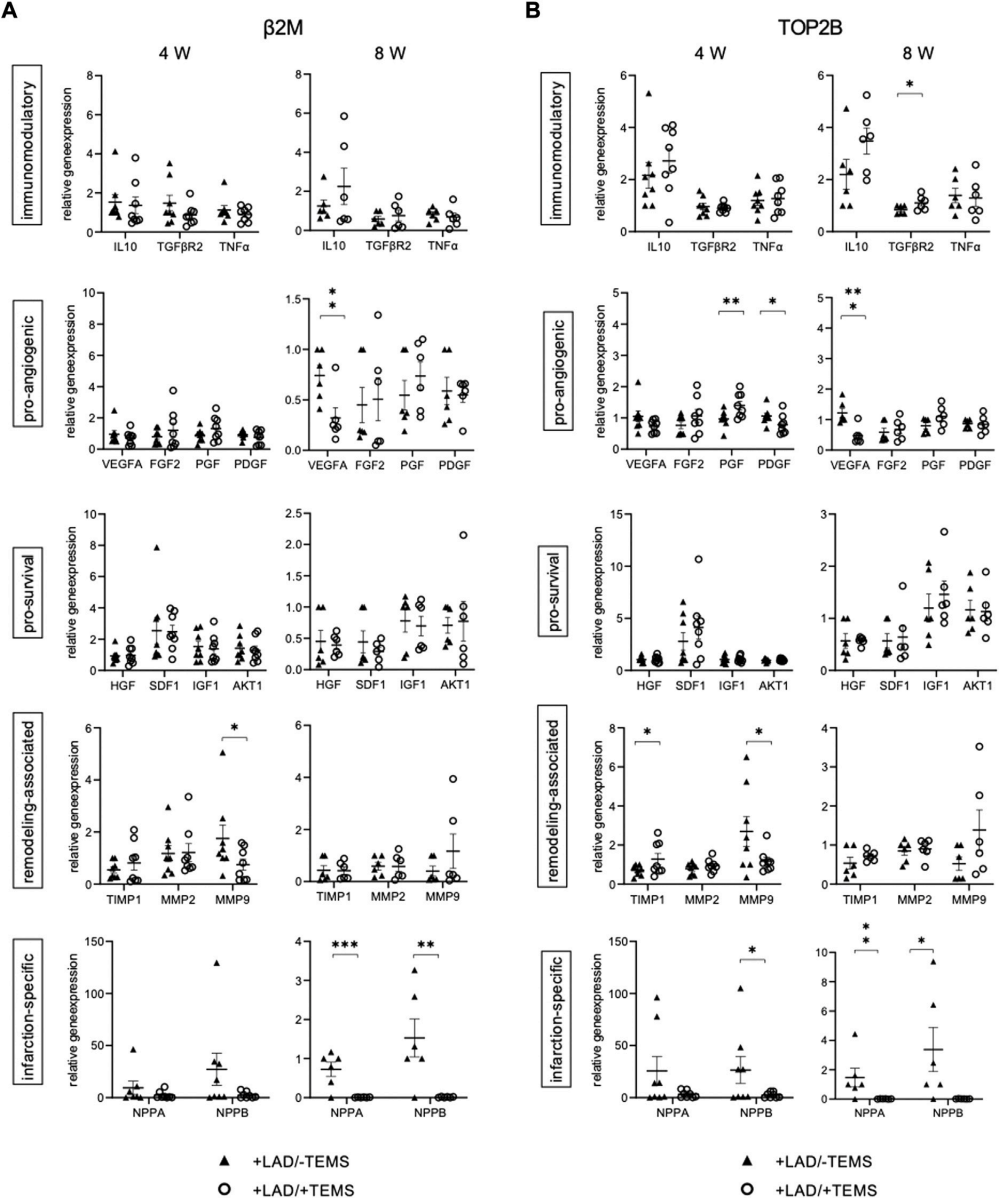
Gene expression of cells infiltrating the TEMS scaffold. Relative gene expression patterns of TEMS samples removed from the native myocardium after either 4 or 8 weeks *in vivo* after myocardial infarction via LAD-ligation (+LAD) or respective sham control (-TEMS). **(A)** Gene expression relative to β2M housekeeping gene. **(B)** Gene expression relative to TOP2B housekeeping gene. Data presented as mean ± SD; *, *p* < 0.05; **, *p* < 0.01; ***, *p* < 0.001.

Interestingly, cells in the TEMS scaffold had a different gene expression pattern depending on whether the cdECM scaffold was placed on healthy or infarcted myocardium (Supplementary Table S3 **,** Figure 6). After 4 weeks *in situ* the TEMS scaffold on infarcted myocardium showed significantly increased expression of pro-angiogenic PGF (1.40 ± 0.36 vs 0.97 ± 0.25; top2b; *p* < 0.05) and reduced PDGFB (0.78 ± 0.29 vs 1.06 ± 0.24; top2b; *p* < 0.04), increased expression of remodeling-associated TIMP1 (1.28 ± 0.78 vs 0.71 ± 0.24; top2b; *p* < 0.05) and reduced expression of MMP9 (1.18 ± 0.54 vs 2.70 ± 2.04; top2b; *p* < 0.05) as well as reduced infarction-related expression of NPPB (2.41 ± 2.44 vs 26.42 ± 34.14; top2b; *p* < 0.05), as compared to the TEMS scaffold placed on non-infarcted myocardium (Figures 6A,B **)**. After 8 weeks *in situ* the TEMS scaffold on infarcted myocardium showed significantly increased expression of immunomodulatory TGFBR2 (0.68 ± 0.16 vs 0.84 ± 0.15; top2b; *p* < 0.05), decreased expression of pro-angiogenic VEGFA (0.02 ± 0.01 vs 1.22 ± 0.33; top2b; *p* < 0.001), as well as reduced infarction-related expression of NPPA (2.41 ± 2.44 vs 1.47 ± 1.41; top2b; *p* < 0.05) and NPPB (0.02 ± 0.01 vs 3.38 ± 3.39; top2b; *p* < 0.05), as compared to the TEMS scaffold placed on non-infarcted myocardium (Figures 6A,B **).**

## Discussion

This proof-of concept study demonstrates that ventricular stabilization after myocardial infarction with a customized, decellularized cardiac ECM-based scaffold (TEMS) significantly alters myocardial gene expression in a rodent LAD-ligation model. Further, orientating analysis shows improved functional echocardiographic parameters as well as biological integration of the TEMS scaffold after 4 and 8 weeks of ventricular stabilization *in vivo.*

In the past, different surgical and experimental approaches have used the concept of cardiomyoplasty in terms of functional left ventricular restoration in order to treat end-stage heart failure. This concept was first introduced in 1985 by Carpentier and Chaques as dynamic cardiomyoplasty, by wrapping the M. latissimus dorsi circumferentially around the heart in order to support systolic contraction (Carpentier and Chachques, 1985). Another surgical approach was the implantation of a mesh-like woven polyester jacket circumferentially around the heart (Acorn CorCap cardiac support device, Acorn Cardiovascular, Inc., St Paul, Minn.) to provide support during diastole (Starling and Jessup, 2004). Although those surgical cardiomyoplasty approaches were used in thousands of patients, they were ultimately abandoned, as outcomes were disappointing with questionable improvement of cardiac hemodynamics and concerns regarding adverse effects such as pericardial constrictions (Acker, 1999; Power et al., 2001). However, one of the main effects of surgical cardiomyoplasty was its ability to prevent adverse left ventricular remodeling, such as attenuation of cardiac enlargement and specific reduction of left ventricular end-diastolic dimensions.

As shown in this study ventricular stabilization via TEMS implantation prevents progressive left ventricular dilatation after myocardial infarction. Rats with supported ventricles via TEMS implantation after LAD-ligation did not only present with significantly smaller LVEDDs after 8 weeks as compared to their controls, but also showed LVEDDs comparable to the values at the time-point of TEMS implantation 14 days after myocardial infarction. Together with the other functional echocardiographic parameters, this demonstrates the potential of the TEMS concept to prevent adverse cardiac remodeling. It is still unclear what triggers this effect. Decellularized cardiac ECM still has a certain stiffness which might offer passive mechanical support during diastole (Brazile et al., 2021), leading to similar effects as with above-mentioned Acorn CorCap device. However, as demonstrated by the orientating histological analysis the TEMS scaffold is not only largely infiltrated by host cells, but also seems to integrate into the scar zone after myocardial infarction. Hence, a biological interaction with the host tissue is likely to play a mechanistic role.

The alteration of the gene expression pattern of the infarcted myocardium depending on whether the LV was supported by TEMS implantation or not, as well as the different gene expression pattern of the cells infiltrating the TEMS scaffold depending on whether it was placed on healthy or infarcted myocardium, seem to demonstrate a high degree of biological interdependency. TEMS implantation seemingly inhibited an up-regulation of infarction-related NPPA and NPPB, while promoting a down-regulation of pro-angiogenic PDGFB and immunomodulatory TGFBR2 and an up-regulation of pro-survival IGF1 and immunomodulatory IL-10 after 4 weeks of ventricular stabilization. Multiple pathophysiological factors converge after myocardial infarction leading to adverse remodeling and ischemic cardiomyopathy (Prabhu and Frangogiannis, 2016). Alteration of gene expression, as shown in this study, might enhance the post myocardial infarction regenerative process attenuating deteriorative effects of cardiac remodeling. For instance, platelet-derived growth factors as well as transforming growth factor-beta are involved in the negative inflammatory and fibrogenic response in the infarcted myocardium (Myocardial Remodeling, 2011; Dobaczewski et al., 2011) while IL-10 is a potent anti-inflammatory agent suppressing the synthesis of proinflammatory cytokines (Fiorentino et al., 1991), and activation of IGF-1 protects from the detrimental effects of myocardial infarction (Troncoso et al., 2014). On the other hand, down-regulation of NPPB, which is mainly secreted by the myocardium due to increased transmural pressure or volume overload and is considered a hallmark for maladaptive LV remodeling, could be a consequence of the TEMS capacity to prevent LV dilatation 4 weeks after myocardial infarction (Fu et al., 2018). Further, significant differences in gene expression could still be demonstrated 8 weeks after TEMS implantation with an up-regulation of remodeling-associated MMP9 and pro-angiogenic PDGFB and down-regulation of FGF2. Together with the echocardiographic findings demonstrating improved functional parameters and a further decrease in LV dimensions, our results advocate for beneficial long-term effects of ventricular stabilization via a cdECM scaffold.

Since in this proof-of-concept study only an orientating histological analysis of the explanted TEMS specimens was conducted, it remains unclear which cells infiltrate the TEMS scaffold and what role they play in the observed effects with regards to cardiac remodeling and improved cardiac function. Interestingly though, gene expression patterns of those cells significantly varied depending on whether the TEMS was placed on infarcted or healthy myocardium. During implantation the TEMS scaffold is only loosely placed on the epicardium, therefore the cdECM-scaffold seems to inherently attract resident cells, which possess the ability to integrate the scaffold into the myocardial tissue especially in the region of infarcted myocardium. Recent research efforts are increasingly demonstrating the importance of epicardial-myocardial signaling and epicardial stem cells in cardiac repair (Pellegrini et al., 2020). Therefore, a cdECM scaffold such as the TEMS might provide an ideal environment for recruitment, activation and differentiation of epicardial cells, which might enhance the regenerative capacity of the heart (Moroni and Mirabella, 2014). Targeted biofunctionalization of cdECM with specific biological cues, could further modulate TEMS-cell interaction by specific cell recruitment and activation enhancing the TEMS effects (Aubin et al., 2016).

Further, cardiac-derived decellularized ECM scaffolds can be repopulated *ex vivo* offering the possibility to enhance the TEMS concept with appropriate pro-regenerative cell populations potentially further increasing cardiac repair (Aubin et al., 2013b; Hülsmann et al., 2013). This would follow the cellular cardiomyoplasty approach, which aims at employing stem cell technologies in order to induce cardiac repair (Lamb et al., 2013). Although in the past cellular cardiomyplasty has shown the potential to decrease fibrosis of infarcted myocardium, to prevent adverse post-ischemic remodeling and to some extent improve systolic cardiac function, clinical outcomes have been disappointing so far, with low rates of cell survival and shortage of donor cells limiting further translation into the routine clinical practice (Broughton et al., 2018). Here, the TEMS platform could offer a promising delivery strategy that could be combined with induced pluripotent stem cell (iPSC) technology, which now allows for the derivation of large numbers of autologous cardiomyocytes from patient-specific iPSCs (Duran et al., 2018). In fact, engineered heart tissue patches generated from fibrinogen-thrombin and human iPSCs have already shown potential for remuscularization and improvement of cardiac function in a pre-clinical large animal model (Querdel et al., 2021).

In conclusion, ventricular stabilization of the heart via a cdECM-scaffold in form of a TEMS stimulates myocardial regeneration, attenuates cardiac remodeling and improves cardiac hemodynamics in a rodent model. Hence, the TEMS approach might be a promising therapy strategy for inducing cardiac regeneration in diseased hearts following a novel bioactive cardiomyoplasty concept. However, further studies are warranted in order to confirm those findings and translate the concept into a clinical setting.

### Limitations

As this is a first proof-of-concept study several limitations remain. Cardiac functional parameters are based solely on echocardiographic parameters and need to be validated with more sensitive analysis such as MRI or invasive catherization. Although gene expression analysis was mostly consistent between both housekeeping genes, there is still a large biological variability within the reduced sample size. Further analysis in order to elucidate on the mechanism behind the observed biological effects and to characterize infiltrating cells are warranted. As this study was conducted in a rodent animal model, results should be considered with caution when applied to humans.

## Data Availability

The original contributions presented in the study are included in the article/supplementary material, further inquiries can be directed to the corresponding author.
