# Supplementary material for: Ventricular stabilization with a customized decellularized cardiac ECM-based scaffold after myocardial infarction alters gene expression in a rodent LAD-ligation model
Source: Front Bioeng Biotechnol. 2022 Sep 23;10:896269. doi: 10.3389/fbioe.2022.896269 (PMC9537373; doi:10.3389/fbioe.2022.896269)
Supplement: Supplementary file 1 [file DataSheet1.docx]

# Supplementary material

**S-Tab. 1.: Analyzed genes.**

| **gene group** | **specific gene** | **primer** |
| --- | --- | --- |
| **immunomodulatory** | Interleukin -10  (IL 10) | F: GACGCTGTCATCGATTTCTCCC  R: GCCTTGTAGACACCTTTGTCTTG |
|  | Tumor necrosis factor-α  (TNF α) | F: GCT CCC TCT CAT CAG TTC CA  R: GCT TGG TGG TTT GCT ACG AC |
|  | Transforming growth factor, beta receptor II  (TGFBR2) | F: CCCAAGTCGGTTAACAGCGA  R: ACAGTGTCACGTCGCAAAAC |
| **pro-angiogenic** | Vascular endothelial growth factor A  (VEGFA) | F: CTGGACCCTGGCTTTACTGC  R: ACTTCACCACTTCATGGGCTT |
|  | Fibroblast growth factor 2  (FGF2) | F: CGGCTCTACTGCAAGAACGG  R: TGTAGTTTGACGTGTGGGTCG |
|  | Placental growth factor  (PGF) | F: CTTGGTCCTCTGTTCCACCC  R: ACAGTAGACTGGGTCCCCTC |
|  | Platelet-derived growth factor beta polypeptide  (PDGFB) | F: GAGAGTGTGGGCAGGGTTAT  R: CATCGAGACAGACGGACGAG |
| **pro-survival** | Hepatocyte growth factor  (HGF) | F: GAACACAGCTTTTTGCCTTCG  R: CCCCTCGAGGATTTCGACAG |
|  | Stromal cell-derived factor 1  (SDF1) | F: ACAAGTGTGCATTGACCCGA  R: GCGTCTGACTCACACCTCTC |
|  | Insulin-like growth factor 1  (IGF1) | F: CCACACTGACATGCCCAAGA  R: GTACTTCCTTTCCTTCTCCTTTGC |
|  | V-akt murine thymoma viral oncogene homolog 1/ Proteinkinase B α  (AKT1) | F: CTACGGTGCGGAGATTGTGT  R: GTTCTCCAGCTTGAGGTCCC |
| **remodeling-associated** | Matrix metalloproteinase 2  (MMP2) | F: AGAAGGCTGTGTTCTTCGCA  R:GGTCAGTGGCTTGGGGTATC |
|  | Matrix metalloproteinase 9  (MMP9) | F: AGAGACACGCTAGAGCAGAT  R: AGCAACAAGAGGATGCCAGA |
|  | Tissue inhibitor of metalloproteinase 1  (TIMP1) | F: AGAGACACGCTAGAGCAGAT  R: AGCAACAAGAGGATGCCAGA |
| **infarction-specific** | Natriuretic peptide A  (NPPA) | F: CTGCTTCGGGGGTAGGATTG  R: TTCGGTACCGGAAGCTGTTG |
|  | Natriuretic peptide B  (NPPB) | F: AGCTGCTTTGGGCAGAAGAT  R: AAAACAACCTCAGCCCGTCA |
| **housekeeping genes** | Beta-2-microglobulin  (β2m) | F: ACTGAATTCACACCCACCGA  R: TACATGTCTCGGTCCCAGGT |
|  | Topoisomerase-II-beta  (TOP2B) | F: ATTGGGACTGGATGGGCTTG  R: GCATCCGCCTGACATTGTTC |

**S-Tab. 2: Relative changes in gene expression of the anterior left ventricular myocardial wall after LAD-ligation.**

| **gene group** | | **relative changes in gene expression*** | | | |
| --- | --- | --- | --- | --- | --- |
|  |  | **4 weeks** | | **8 weeks** | |
| **housekeeping** | | TOP2B | β2M | TOP2B | β2M |
| **immunomodulatory** | IL 10 | ↑ | ↑ | ↑ | ↑ |
|  | TNF α | ↑ | ↑ | ↓ | ↓ |
|  | TGFBR2 | ↑*** | ↓ | ↑ | → |
| **pro-angiogenic** | VEGFA | ↓** | ↓^*^ | ↓* | ↓* |
|  | FGF2 | ↑*** | ↑* | ↑** | ↑ |
|  | PGF | ↑*** | ↑ | ↑* | ↑ |
|  | PDGFB | ↓ | ↓ | ↓ | ↓ |
| **pro-survival** | HGF | ↑*** | ↑*** | ↑* | ↑* |
|  | SDF1 | ↑** | ↑* | ↓ | ↓ |
|  | IGF1 | ↑** | ↑ | ↑* | ↑* |
|  | AKT1 | ↓* | ↓* | ↓* | ↓** |
| **remodeling-associated** | MMP2 | ↑*** | ↑* | ↑ | ↑ |
|  | MMP9 | ↑** | ↑** | ↑ | ↑ |
|  | TIMP1 | ↑** | ↑** | ↑^**^ | ↑^*^ |
| **infarction-specific** | NPPA | ↑*** | ↑*** | ↑^**^ | ↑^**^ |
|  | NPPB | ↑ | ↑ | ↑ | ↑ |

*Relative changes to anterior left ventricular myocardial wall of respective sham control group. *, p<0.05; **, p<0.01; ***, p<0.001

**S-Tab. 3: Relative changes in gene expression of the cells infiltrating the TEMS scaffold after implantation on infarcted hearts as compared to healthy hearts.**

| **gene group** | | **relative changes in gene expression** | | | |
| --- | --- | --- | --- | --- | --- |
|  |  | **4 weeks** | | **8 weeks** | |
| **housekeeping** | | TOP2B | β2M | TOP2B | β2M |
| **immunomodulatory** | IL 10 | ↑ | ↓ | ↑ | ↑ |
|  | TNF α | ↑ | ↓ | ↓ | ↓ |
|  | TGFBR2 | ↓ | ↓ | ↑* | ↑ |
| **pro-angiogenic** | VEGFA | ↓ | ↓ | ↓*** | ↓** |
|  | FGF2 | ↑ | ↑ | ↑ | ↑ |
|  | PGF | ↑** | ↑ | ↑ | ↑ |
|  | PDGFB | ↓* | ↓ | ↓ | ↓ |
| **pro-survival** | HGF | ↑ | ↑ | ↓ | ↓ |
|  | SDF1 | ↑ | ↓ | ↑ | ↓ |
|  | IGF1 | ↑ | ↓ | ↑ | ↓ |
|  | AKT1 | ↑ | ↓ | ↓ | ↑ |
| **remodeling-associated** | MMP2 | ↑ | ↑ | ↑ | ↓ |
|  | MMP9 | ↓* | ↓* | ↑ | ↑ |
|  | TIMP1 | ↑* | ↑ | ↑ | ↓ |
| **infarction-specific** | NPPA | ↓ | ↓ | ↓** | ↓*** |
|  | NPPB | ↓* | ↓ | ↓* | ↓** |

*, p<0.05; **, p<0.01; ***, p<0.001
